# Impact of Ocean Acidification on the Energy Metabolism and Antioxidant Responses of the Yesso Scallop (*Patinopecten yessoensis*)

**DOI:** 10.3389/fphys.2018.01967

**Published:** 2019-01-21

**Authors:** Huan Liao, Zujing Yang, Zheng Dou, Fanhua Sun, Sihua Kou, Zhengrui Zhang, Xiaoting Huang, Zhenmin Bao

**Affiliations:** ^1^MOE Key Laboratory of Marine Genetics and Breeding, College of Marine Life Sciences, Ocean University of China, Qingdao, China; ^2^College of Animal Biotechnology, Jiangxi Agricultural University, Nanchang, China; ^3^Laboratory for Marine Fisheries Science and Food Production Processes, Qingdao National Laboratory for Marine Science and Technology, Qingdao, China

**Keywords:** ocean acidification, energy metabolism, oxidative stress, physiological response, scallop

## Abstract

Ocean acidification (OA), which is caused by increasing levels of dissolved CO_2_ in the ocean, is a major threat to marine ecosystems. Multiple lines of scientific evidence show that marine bivalves, including scallops, are vulnerable to OA due to their poor capacities to regulate extracellular ions and acid-based status. However, the physiological mechanisms of scallops responding to OA are not well understood. In this study, we evaluated the effects of 45 days of exposure to OA (pH 7.5) on the energy metabolism and antioxidant capability of Yesso scallops. Some biochemical markers related to energy metabolism (e.g., content of glycogen and ATP, activity of ATPase, lactate dehydrogenase, glutamate oxaloacetate transaminase, and glutamate-pyruvate transaminase), antioxidant capacity (e.g., reactive oxygen species level, activity of superoxide dismutase, and catalase) and cellular damage (e.g., lipid peroxidation level) were measured. Our results demonstrate that the effects of the reduced pH (7.5) on scallops are varied in different tissues. The energy reserves are mainly accumulated in the adductor muscle and hepatopancreas. Yesso scallops exhibit energy modulation by increasing lactate dehydrogenase activities to stimulate anaerobic metabolism. The highly active Na^+^/K^+^-ATPase and massive ATP consumption in the mantle and gill indicate that a large amount of energy was allocated for the ion regulation process to maintain the acid-base balance in the reduced-pH environment. Moreover, the increase in the reactive oxygen species level and the superoxide dismutase and catalase activities in the gill and adductor muscle, indicate that oxidative stress was induced after long-term exposure to the reduced-pH environment. Our findings indicate that the effects of OA are tissue-specific, and physiological homeostasis could be modulated through different mechanisms for Yesso scallops.

## Introduction

Increasing amounts of anthropogenic CO_2_ from the atmosphere dissolved in seawater has changed the overall seawater chemistry with a net increase in hydrogen (H^+^) and bicarbonate ions (HCO_3_^−^) and a decrease in carbonate ions (CO_3_^2−^), which has already resulted in a 0.1 unit decline in seawater pH since the Industrial Revolution (Caldeira and Wickett, 2003, 2005; Sabine et al., 2004; Feely, 2011; Perez et al., 2018). These changes are known as ocean acidification (OA). According to the prediction of the Intergovernmental Panel on Climate Change (IPCC), the pH of surface seawater could further decrease to 7.8 and 7.4 by the years 2100 and 2300, respectively (Caldeira and Wickett, 2003; Stocker, 2013). Many studies have confirmed that the predicted changes of seawater pH will have widespread negative effects on the structure, function and fitness of marine ecosystems (Feely, 2011; Uthicke et al., 2013). The variety of responses within and between taxa suggest that OA is a driver for substantial change in ocean ecosystems this century, potentially leading to long-term shifts in species composition (Pörtner and Farrell, 2008; Wittmann and Pörtner, 2013). As a result, understanding the potentials of maintaining physiological homeostasis will help predict biological stability and changes to rapid anthropogenic modifications of ecosystems and geosystems (Somero, 2010; Applebaum et al., 2014).

Marine bivalves, such as oyster, mussel, clam and scallop, are widely distributed, and they are economically and ecologically important (Gazeau et al., 2013; Kroeker et al., 2013). Scientific literature has revealed that most mollusk species are vulnerable to OA (Pörtner and Farrell, 2008; Wittmann and Pörtner, 2013). To regulate physiological homeostasis, energy is required in response to the environmental changes. Metabolic energy demands under acidification stress may exceed the energy supply from food or/and accrued energy resources, which might lead to a lack of adenosine triphosphate (ATP) to sustain routine metabolism (Lannig et al., 2010). However, some mollusks, such as bivalve fingernail clam *Sphaerium occidentale* and the cephalopod *Nautilus pompilius*, can overwhelm the capacity of systemic functions or inhibit the metabolic rate to conserve energy and to extend the survival time (Guppy, 1999). Recent studies have shown that modest increases in dissolved CO_2_ (<1200 μatm) have little effect on metabolic rates, whereas extremely high levels of dissolved CO_2_ (>5000 μatm) could depress metabolic rates (Michaelidis et al., 2005; Beniash et al., 2010; Hendriks et al., 2010; Thomsen and Melzner, 2010; Zhao et al., 2017b). As shown by Cao et al. (2018), Pacific oyster (*Crassostrea gigas*) modulates energy sources by inhibiting aerobic energy metabolism, stimulating anaerobic metabolism, and increasing glycolytic enzyme activity following exposure to a reduced pH value of 7.6 for 28 days. The clams *(Ruditapes philippinarum*) were reported to be able to maintain/regulate their physiological status and biochemical performance under reduced pH (7.3) for 28 days (Velez et al., 2016). However, in blue mussel *Mytilus edulis*, energy and protein metabolism were strongly affected by elevated *p*CO_2_ (1,120, 2,400, or 4,000 μatm after 2 months of treatment) (Hüning et al., 2013). In addition, some recent transcriptomic analyses have indicated that marine bivalves implement a compensatory acid-base mechanism, metabolic depression and positive physiological responses to mitigate the effects of OA (Goncalves et al., 2016, 2017a,b; Li et al., 2016a,b; Peng et al., 2017).

Oxidative stress, the production and accumulation of reactive oxygen species (ROS), is an important component of the stress response for marine organism when exposed to environmental change (Lesser, 2006). In response to oxidative stress, a wide set of antioxidant systems including superoxide dismutase (SOD) and catalase (CAT) have evolved in marine organisms (Lesser, 2006). The oxidative stress response is prevalent in marine bivalves when exposed to elevated CO_2_ levels. As shown in the mussel *M. galloprovincialis*, the oxidative status was negatively affected by reduced pH with an increase in antioxidant enzymes activity and ROS overproduction (Freitas et al., 2017). In the mussel *M. coruscus*, most biochemical indexes [SOD, CAT, glutathione peroxidase (GPx), acid phosphatase (ACP), and alkaline phosphatase (ALP)] measured in gills and hemocytes were increased when the mussels were subjected to reduced pH (Huang et al., 2018). Nevertheless, no persistent oxidative stress signal was observed during long-term (8–15 weeks) exposure to moderately elevated CO_2_ (∼800 ppm CO_2_) in hard shell clam *Mercenaria mercenaria* and eastern oyster *C. virginica* (Matoo et al., 2013). In addition, some studies have demonstrated that the key target genes associated with antioxidant defenses (ecSOD, catalase, and peroxiredoxin 6) were susceptible to OA in marine bivalves (Goncalves et al., 2016, 2017a,b; Li et al., 2016a,b; Wang et al., 2016).

All of these studies indicated that the metabolic effects and antioxidant responses varied among different marine species under elevated dissolved CO_2_ seawater (Dupont et al., 2010; Hendriks et al., 2010; Kroeker et al., 2010). However, there was very limited information about the effects of OA on scallops (Benedetti et al., 2016; Haider et al., 2016; Nardi et al., 2018). The Yesso scallop (*Patinopecten yessoensis*) is an economically important shellfish in China, and the production is approximately 240,000 tons in 2016 (data from 2017 China Fishery Statistical Yearbook). The present study aimed to systematically assess the metabolic effects and antioxidant responses of Yesso scallops under present and future predicted sea surface pH. After 45 days of exposure to two pH levels (8.0 and 7.5), we measured some biochemical markers to determine the energy metabolism levels [assessed by the activity of ATPase, lactate dehydrogenase (LDH), glutamate oxaloacetate transaminase (GOT) and glutamate-pyruvate transaminase (GPT), content of glycogen (GLY) and ATP]; antioxidant capacity (ROS, CAT, and SOD); and cellular damage [measured by the lipid peroxidation (LPO) level] of four tissues (adductor muscle, mantle, gill, and hepatopancreas). The findings of the current study can improve our understanding of the physiological mechanisms involved in regulating energy metabolism and antioxidant responses in marine bivalves when exposed to elevated CO_2_ levels.

## Materials and Methods

### Experimental Animals Collection and Acclimation

Samples of 2-year-old Yesso scallops were collected from Haiyi Seeds Co., LTD, in Shandong province, China, in September 2017. The scallops without shell damage were transported on ice to the laboratory and acclimated for 1 week in a recirculated aquarium system equipped with a filtering system (pH 8.0, temperature 18°C, and salinity 30.2‰). During the acclimation period, all scallops were fed twice daily with *Nitzschia closterium* (40,000 cells /animal), and the seawater was changed daily.

### Ocean Acidification Stimulation and Seawater Chemistry Analysis

After 1 week of acclimation, the healthy scallops were randomly divided into two groups and placed in two different environments: the control group at a pH of 8.0 (current concentration of *p*CO_2_) and the experimental group at a pH of 7.5 (representing the predicted water surface pH level of the twenty-first century) (Caldeira and Wickett, 2003; Stocker, 2013). For each treatment, there were three replicates of ∼50-L tanks, holding ten scallops each. The reduced pH value (pH 7.5) was maintained by vigorously bubbling ambient air or a CO_2_ gas mixture. The CO_2_ gas mixture was obtained by mixing CO_2_-free air and pure CO_2_ gas at known flow rates using flow controllers (Zhao et al., 2017a). The pH of seawater was monitored using a pH meter (Sartorius PB-10) to ensure no substantial change in pH during the experiment. The other seawater parameters including salinity, temperature and dissolved oxygen (DO) were measured daily by a multi-parameter water quality meter (HORIBA U-52, Japan). The total alkalinity (TA) was monitored once a week by potentiometric titration (Anderson and Robinson, 1946). The carbonate system parameters were calculated using CO2SYS software (Pierrot et al., 2006), and the seawater parameters for the experimental exposures are given in Table 1.

**Table 1:** Water chemistry parameters during 45 days of incubation of *P. yessoensis*.

Treatment	Measured parameters				Calculated parameters		
	pH_NBS_	T (°C)	Sal (‰)	TA(μmol/kg)	*p*CO2(μatm)	Ωc	Ωa
Control	8.01 ± 0.02	18.35 ± 0.21	30.27 ± 0.25	2282.67 ± 149.70	546.66 ± 71.81	3.5 ± 0.33	2.17 ± 0.21
CO_2_-incubation	7.51 ± 0.01	18.66 ± 0.30	30.47 ± 0.17	2260.1 ± 66.10	2217.91 ± 87.75	1.16 ± 0.06	0.72 ± 0.03

*pH_NBS_, pH calibrated with National Bureau of Standard scale; T, temperature; Sal, salinity; TA, total alkalinity; pCO_2_, partial pressure of CO_2_; Ωc and Ωa, saturation state of calcite and aragonite, respectively. Data are presented as the means ± standard deviations.*

### Biochemical Assays

After 45 days of experiments, six scallops per treatment were collected at random. Four tissues, including the adductor muscle, mantle, gill and hepatopancreas were collected from each specimen and stored at −80°C until further analyses. For GLY content determination, the tissues were added to an alkaline solution (1:3, w:v) in boiling water for 20 min, aiming to destroy all components except GLY. For the remaining biochemical markers, the tissues were added to an ice-cold physiological saline solution (1:9, w:v) and homogenized by ultrasonic treatment (0°C, 360 Watt). The homogenization was then centrifuged for 10 min at 2500 rpm and 4°C. The supernatants were stored at −80°C or immediately used to determine the content of ROS and ATP, the LPO levels, and the activities of SOD, CAT, GOT, GPT, LDH, and ATPase.

All of the enzymatic activities and biochemical assays were analyzed following the instructions of kits from the Nanjing Jiancheng Bioengineering Institute. Optical density values were measured using a microplate reader (Infinite 200 pro, Tecan). The results of the enzymatic activities were expressed as the unit per gram or unit per milligram of protein (U/g or U/mg protein).

Reactive oxygen species production was quantified using 2,7-dichlorofluorescin diacetate (DCFH-DA) treatment (Eruslanov and Kusmartsev, 2010). DCFH-DA is a non-polar dye, and it is converted into the polar derivative DCFH by cellular esterases that are non-fluorescent but would be oxidized by ROS to form highly fluorescent DCF (Burow and Valet, 1987). The fluorescence intensity was positively correlated with the ROS content, which was measured at the excitation and emission wavelengths of 500 and 530 nm, respectively. The results were referred to the standard curve drawn by positive control of the reactive oxygen donor with gradient concentrations.

The LPO levels were determined by measuring malondialdehyde (MDA). The quantitative measurement of MDA was based on the reaction of MDA and thiobarbituric acid (TBA). The reaction product MDA-TBA2 was measured at 532 nm (Janero, 1990).

The SOD activity was measured by the WBT-1 method (Peskin and Winterbourn, 2000). The tetrazolium salt WST-1 was converted into water-soluble WST-1 formazan by a superoxide radical generated by the conversion of xanthine to uric acid and hydrogen peroxide (H_2_O_2_) by xanthine oxidase. Meanwhile, the existence of SOD reduced the concentrations of the superoxide anion radical and thereby lowered the formation rate of WST-1. The absorbance of the final product was measured at 450 nm after 20 min of incubation at 37°C. The results are expressed in unit per milligram of protein (U/mg protein). One unit of SOD activity corresponded to the enzyme quantity when the SOD inhibition rate reached 50% under the assay conditions.

The CAT activity was determined on the basis of the ammonium molybdate method (Góth, 1991). CAT acted on catalyzing the H_2_O_2_ degradation reaction, which was terminated by adding ammonium molybdate. Moreover, ammonium molybdate and H_2_O_2_ reacted rapidly in forming a yellow complex, which was measured at 405 nm. One unit of CAT activity was defined as the degradation of 1 μmol H_2_O_2_ per second per milligram of tissue protein.

The GLY content was quantified according to the anthrone colorimetric method (Carroll et al., 1956). After treatment in concentrated sulfuric acid, GLY dehydrated and produced glycolaldehyde. Subsequently, the production was reacted with anthrone and formed blue compounds, which contained a maximum absorption peak at a wavelength of 620 nm.

The LDH activity was measured by the trace enzyme labeling method (Weeks and Johnson, 1977), which is based on the function of LDH in catalyzing the formation of pyruvate. The pyruvate was reacted with 2, 4-dinitrophenylhydrazine in a terminal indicator reaction to form the characteristic brown color of the corresponding 2, 4-dinitrophenylhydrazone pyruvate, which was measured colorimetrically at 440 nm. One unit of LDH activity was defined as the formation of 1 μmol pyruvate per 15 min per gram protein.

The ATP content was determined according to the phosphomolybdic acid colorimetry method (Chen and Sun, 2002). It is an indirect method for determining the ATP level. In brief, creatine kinase was added to catalyze the formation of phosphocreatine from ATP and creatine, and then the product was detected at 636 nm.

The ATPase activity was quantified following the protocol of the ATP enzyme test kit (Na^+^/K^+^-ATPase and Ca^2+^/Mg^2+^-ATPase) from Nanjing Jiancheng Bioengineering Institute, which was based on ATPase function in decomposing ATP to produce ADP and inorganic phosphorus. The activity of ATPase was judged by the amount of inorganic phosphorus, which was interacted with a phosphorus-fixing agent for 30 min at 37°C. The absorbance was measured at 660 nm when the product cooled to room temperature, and one unit of ATPase was defined as the product of 1 μmol inorganic phosphorus per hour per milligram of protein.

The GPT and GOT activities were quantified following the method of Reitman and Frankel (1957). The activity of GPT or GOT was measured by mixing tissue suspension with the corresponding substrate. Both enzymes were acted on catalyzing the transamination of substrate and the formation of pyruvate. After a 30-min interaction at 37°C, 2, 4-dinitrophenylhydrazine solution was added to stop the transamination and reacted with pyruvate for 20 min at 37°C. Subsequently, the reaction liquid was mixed with NaOH solution for 15 min at room temperature. The optical density was read at 505 nm. The results were referred to the standard curve drawn by the pyruvate standard solution with gradient concentrations and expressed in unit per gram of protein.

### Statistical Analysis

The statistical analyses of the data were preformed using SPSS software (version 20.0). The data collected from this study are expressed as the means ± standard deviation (*N* = 6). A one-way analysis of variance (ANOVA) and Tukey’s test were used to differentiate between the means. Tests were considered significant at *p* < 0.05.

## Results

**FIGURE 1:**
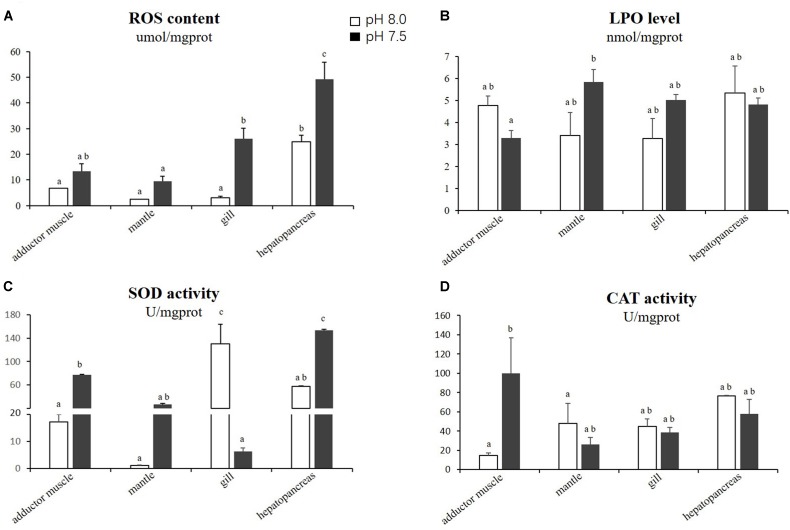
Oxidative stress-related parameters in four tissues of *P. yessoensis* exposed to different pH levels (7.5 and 8.0). **(A)** ROS. **(B)** LPO. **(C)** SOD. **(D)** CAT. Each bar represents the means ± standard deviations (*n* = 6). Different letters indicate significant differences among different treatments (*p* < 0.05).

The levels of ROS, LPO, SOD and CAT in the adductor muscles, mantles, gills and hepatopancreas of scallops exposed to reduced-pH seawater and ordinary seawater (control) are illustrated in Figure 1. The levels of ROS in all tested organs were generally higher in the scallops exposed to reduced-pH seawater compared with the control group. The results of the statistical analyses demonstrate that the ROS levels in the gills and hepatopancreas were significantly higher (*p* < 0.05) in the scallops exposed to reduced-pH seawater than in the control group. For the LPO levels, no significant difference (*p* > 0.05) was observed in all organs between the scallops exposed to reduced-pH seawater and the control group. The SOD activities in the adductor muscles and hepatopancreas were significantly higher (*p* < 0.05) in the scallops exposed to reduced-pH seawater than in the control group. However, the SOD activities in the gills was significantly lower (*p* < 0.05) in the scallops exposed to reduced-pH seawater (6.19 ± 0.46 U mgprot^−1^) than in the control group (130.06 ± 33.73 U mgprot^−1^). For the CAT activities, the scallops exposed to reduced-pH seawater demonstrated significantly higher (*p* < 0.05) CAT activity in the adductor muscles (99.96 ± 36.95 U mgprot^−1^) compared with the scallops in the control group (14.58 ± 2.80 U mgprot^−1^).

**FIGURE 2:**
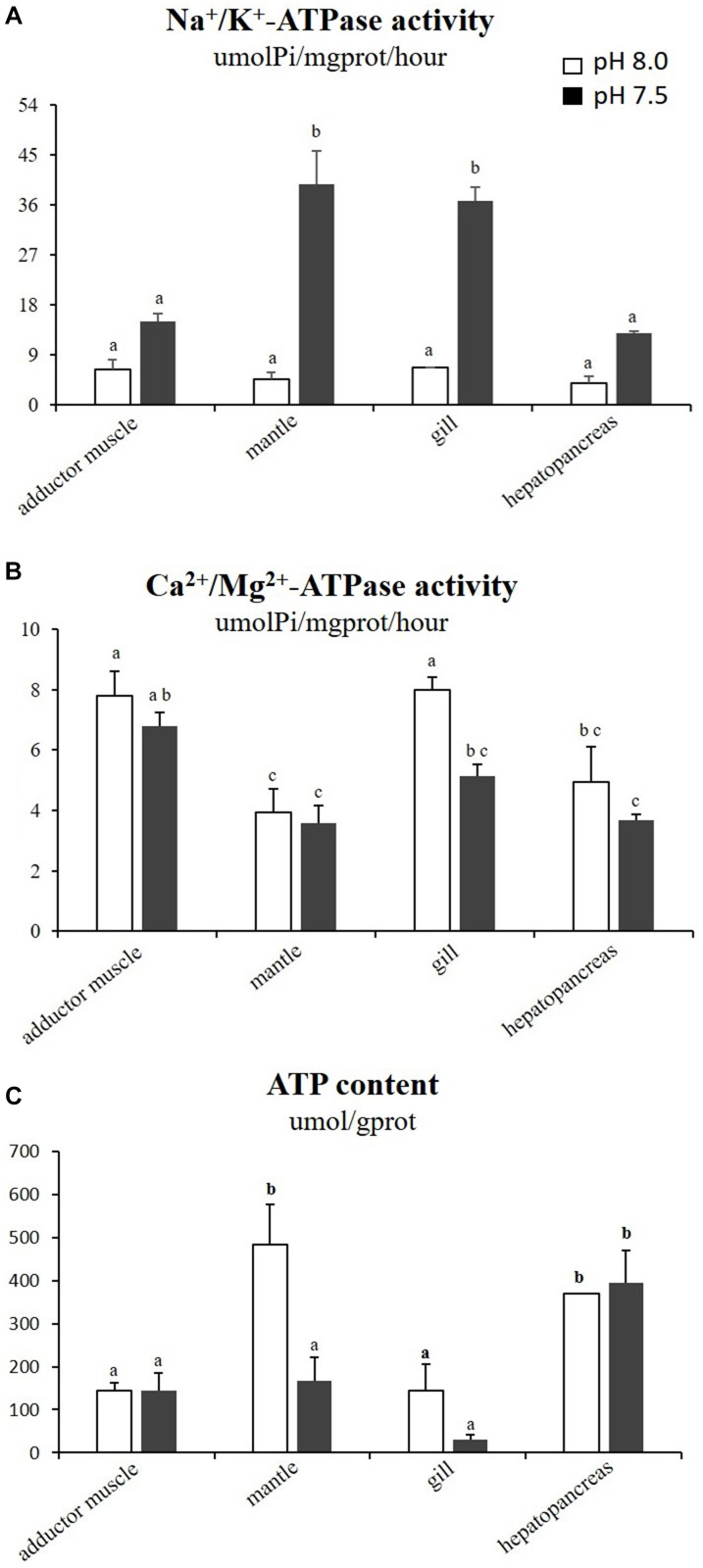
Activities of Na^+^/K^+^-ATPase **(A)**, Ca^2+^/Mg^2+^-ATPase **(B)**, and ATP content **(C)** in four tissues of *P. yessoensis* exposed to different pH levels (7.5 and 8.0). Each bar represents the means ± standard deviations (*n* = 6). Different letters indicate significant differences among different treatments (*p* < 0.05).

The effects of reduced pH on different types of ATPase were varied in the four tissues (Figures 2A,B). The activities of Na^+^/K^+^-ATPase in the mantles and gills of the scallops exposed to reduced-pH seawater were significantly higher (*p* < 0.05) than in the respective organs of the scallops in the control group. The Ca^2+^/Mg^2+^-ATPase activity in the gills of the scallops exposed to reduced-pH seawater (5.13 ± 0.39 μmolPi mgprot^−1^ h^−1^) were significantly lower (*p* < 0.05) than in the gills of the scallops in the control group (7.98 ± 0.42 μmolPi mgprot^−1^ h^−1^). For ATP contents, the scallops exposed to reduced-pH seawater demonstrated significantly lower (*p* < 0.05) ATP contents in the mantles (168.13 ± 52.79 μmol gprot^−1^) compared with the scallops in the control group (483.86 ± 91.41 μmol gprot^−1^) (Figure 2C).

**FIGURE 3:**
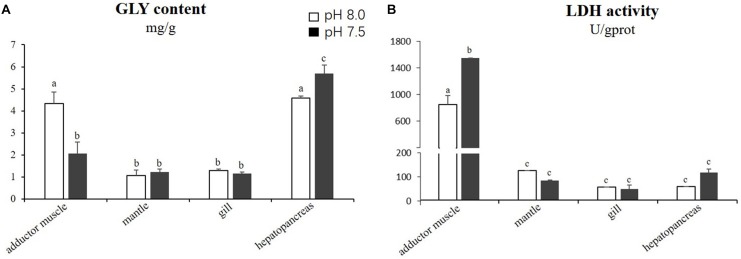
Energy related parameters: GLY content **(A)** and LDH activity **(B)** in four tissues of *P. yessoensis* exposed to different pH levels (7.5 and 8.0). Each bar represents the means ± standard deviations (*n* = 6). Different letters indicate significant differences among different treatments (*p* < 0.05).

The results of the statistical analyses demonstrated that the GLY content in the adductor muscles was significantly lower (*p* < 0.05) in the scallops exposed to reduced-pH seawater (2.06 ± 0.52 mg g^−1^) compared with the control group (4.32 ± 0.53 mg g^−1^). However, the GLY content in the hepatopancreas was significantly higher (*p* < 0.05) in the scallops exposed to reduced-pH seawater (5.70 ± 0.38 mg g^−1^) compared with the control group (4.58 ± 0.09 mg g^−1^) (Figure 3A). The LDH activities in the adductor muscles of the scallops exposed to reduced-pH seawater (1565.31 ± 0.01 U gprot^−1^) were significantly higher (*p* < 0.05) than in the adductor muscles of the scallops in the control group (861.53 ± 134.71 U gprot^−1^) (Figure 3B).

**FIGURE 4:**
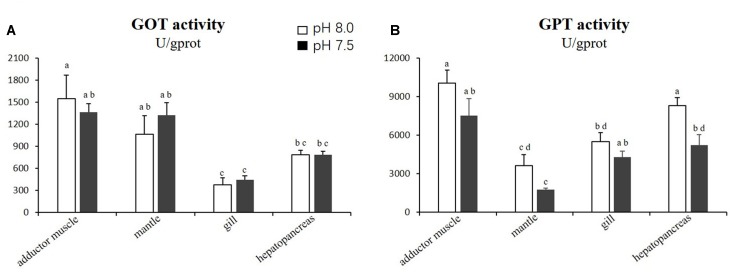
Activities of GOT **(A)** and GPT **(B)** in four tissues of *P. yessoensis* exposed to different pH levels (7.5 and 8.0). Each bar represents the means ± standard deviations (*n* = 6). Different letters indicate significant differences among different treatments (*p* < 0.05).

For the GOT and GPT activities, no significant difference (*p* > 0.05) was observed in the adductor muscles, mantles and gills between the scallops exposed to reduced-pH seawater and the control group. However, the GPT activity in the hepatopancreas was significantly lower (*p* < 0.05) in the scallops exposed to reduced-pH seawater (5227.44 ± 817.48 U gprot^−1^) compared with the control group (8294.14 ± 628.25 U gprot^−1^) (Figure 4).

## Discussion

Ocean acidification caused by increased CO_2_ emission to the atmosphere has been showed in all areas of the ocean including deep sea and coastal waters (Orr et al., 2005; Wittmann and Pörtner, 2013). The impacts of OA are more substantial in coastal waters, where the ecosystem responses to OA could have the most severe implications for mankind (Doney et al., 2007; Feely et al., 2008, 2009, 2010; Wootton et al., 2008; Zhai et al., 2012). Coastal organisms show diverse responses to elevated seawater pCO_2_ (Pörtner and Farrell, 2008; Ries et al., 2009; Hendriks et al., 2010; Wittmann and Pörtner, 2013). The Yesso scallop is an economically important marine shellfish. This species has long been cultivated in the coastal ocean of China, Japan, and Russia (Shumway and Parsons, 2006). However, the potential effects of OA on the physiology of Yesso scallop is still unknown. Thus, the current study aims to evaluate the effects of OA on the scallop based on physiological and biochemical markers.

As a major form of energy storage in cells, GLY supplies energy quickly through glycolysis and oxidative phosphorylation. Adequate glycogen storage in muscle is important to support the optimal movement of muscle (Jensen and Richter, 2012). In the current study, low pH stress resulted in a decrease in glycogen level but an increase in LDH activity in the adductor muscles of scallops. A similar observation has been reported in *C. gigas*, where the GLY level in muscle tissue decreased after exposure to low pH seawater (Lannig et al., 2010). On the other hand, under stressful environments, LDH is an important enzyme that accelerates ATP production through anaerobic processes to maintain energy homeostasis (Strahl et al., 2011). The combined effects of elevated LDH activity and decreased GLY level in muscle tissues suggest that OA accelerates glycolysis, which might be an energy strategy of the Yesso scallop to fuel the high energy demand. The hepatopancreas is another important energy storage organ, and it plays an important role in energy homeostasis of the blood circulation system (Barber and Blake, 1985). The rising in GLY content in the hepatopancreas of the Yesso scallop during reduced-pH exposure suggests that some of the lactic acids from the muscle tissues entered the hepatopancreas through the blood circulation system, where they were then resynthesized to GLY for energetic conservation.

The transaminase GPT plays a key role in mobilizing L-amino acids for gluconeogenesis, and it acts as a link between carbohydrate and protein metabolism under altered physiological, pathological, and induced environmental stress conditions (Ramaswamy et al., 2015). However, the influences of acidification on GPT activity varies by species. A study on the thick shell mussel *M. coruscus* found that the activity of GPT in the gill and digestive gland increased when exposed to reduced-pH seawater (Hu et al., 2015). The current study recorded a contradictory result, where the GPT activity decreased in *P. yessoensis* under the reduced-pH treatment. The hepatopancreas is a major organ for gluconeogenesis. The decrease in GPT activity suggests a reduced turnover of amino acids and protein synthesis in the hepatopancreas of *P. yessoensis*.

In the mantle of *P. yessoensis* after OA exposure, a significant decrease in the ATP level was observed. Nevertheless, the GLY content and LDH activity varied slightly, indicating that glycolysis was not affected. Interestingly, the Na^+^/K^+^-ATPase activity, increased significantly and sharply in the reduced-pH exposure. The enzyme Na^+^/K^+^-ATPase is the main motor of cellular and extracellular acid-base balance and thus acts as an important ion regulatory player, and it may be related to the capability of each species to acclimate to pH fluctuations (Melzner et al., 2009; Velez et al., 2016). The highly active Na^+^/K^+^-ATPase and massive consumption of ATP in the mantle of *P. yessoensis* suggest that a large amount of energy in the mantle was allocated to the ion regulation process to maintain the acid-base balance in the reduced-pH environment. In the mantle of the oyster *C. gigas* following OA-exposure (pH ∼ 7.7) for 1 month, both the ATP content and alanine levels decreased, but the Na^+^/K^+^-ATPase activities were not affected. The authors suggested that alanine is transaminated to pyruvate, which together with ATP is used to build up phosphoenolpyruvate (PEP), with PEP as the substrate entering the gluconeogenetic pathway (Lannig et al., 2010). Other than the mantle, the Na^+^/K^+^-ATPase activities in the gill of *P. yessoensis* were also elevated after OA exposure. On the other hand, the reduced-pH treatment decreased the Ca^2+^/Mg^2+^-ATPase activity without affecting the ATP content in the gills of Yesso scallops. Therefore, it is reasonable to believe that the Yesso scallop could shift the use of its ion regulation mechanism by reducing the Ca^2+^/Mg^2+^-ATPase activity and increasing the Na^+^/K^+^-ATPase activity simultaneously to regulate the acid-base balance under low pH exposure. A similar result has been documented in Atlantic cod (*Gadus morhua*), where a shift in the use of ion regulation mechanisms occurred toward enhanced Na^+^/H^+^-exchange and HCO^3^^−^ transport at high *P*CO_2_ (2200 μatm), paralleled by higher Na^+^/K^+^-ATPase activities, which did not affect the total gill energy consumption and left the whole animal energy budget unaffected (Kreiss et al., 2015).

Reactive oxygen species production is an important internal defense mechanism in bivalves such as *Mytilus edulis*, *Cerastoderma edule*, and *Ensis siliqua* (Wootton et al., 2003; Terahara and Takahashi, 2008). When the ROS content exceeds the antioxidant capacity, the excess ROS may lead to cell oxidative damages, such as DNA damage, membrane lipid damage and enzyme inactivation (Cheng et al., 2004a,b). Increasing ROS content and active peroxidase activities after low pH exposure have been reported in *M. coruscus*, *C. ariakensis*, and *C. gigas* (Bouilly et al., 2006; Gagnaire et al., 2006; Donaghy et al., 2009; Wang et al., 2016; Wu et al., 2016). In the current study, exposure to reduced-pH seawater led to high ROS production in the gill and hepatopancreas of *P. yessoensis.* The gill plays a vital role in respiration, and the hepatopancreas is a major organ for metabolism. These two organs have been shown to be sensitive to environmental stress in bivalves (Farcy et al., 2009; Lockwood et al., 2010). The high ROS content indicates that the oxidative stress occurred in the gill and hepatopancreas of Yesso scallops.

The levels of LPO, SOD, and CAT enzyme activities have been used as biomarkers to assess oxidative stress (Livingstone, 2001; Soldatov et al., 2007; Velez et al., 2016). In our study, the lipid peroxidation level did not change significantly in the reduced-pH group, suggesting that the reduced-pH treatment has little effect on the lipid peroxidation. A similar result was reported in the clam, *R. philippinarum* (Velez et al., 2016). However, the SOD activities were elevated in the adductor muscles and hepatopancreas but decreased in the gill of *P. yessoensis* after exposure to a reduced-pH seawater. The induction of SOD, a primary scavenger of O_2_^−^ generated during stress condition, could inhibit the accumulation of oxygen radicals during exposure to low pH seawater. The weakening SOD activity in the gill tissue could indicate oxidative stress. Furthermore, the increased SOD activity resulted in a high level of H_2_O_2_, which should stimulate CAT activity. In our study, the activity of CAT increased in the adductor muscles, which could have been stimulated by the increased SOD activity.

## Conclusion

The findings of the present study demonstrate that a reduction in seawater pH to 7.5 can potentially interfere with the energetic metabolism and antioxidant responses of scallops. Alteration of the energy demand or reserve has been observed in Yesso scallops after exposure to a reduced-pH environment. The muscle glycogen was decomposed and the activity of LDH increased, leading to suppressed aerobic metabolism and increased anaerobic metabolism. At the same time, the content of glycogen increased in the hepatopancreas, indicating that scallops were able to reserve some energy when exposed to a reduced-pH environment. In addition, the high Na^+^/K^+^-ATPase activities in the mantle and gill suggest that energy was allocated to ion transport in maintaining the intracellular and extracellular acid-base balance. Moreover, increased oxidative stress was also induced after long-term exposure to reduced-pH seawater. Overall, our findings are useful for a better understanding of the biochemical strategies and biological limitations of scallop if the ocean acidification worsens as predicted.

## Author Contributions

HL, XH, and ZB conceived and designed the experiments, analyzed the data, and drafted the manuscript. HL, ZY, ZD, FS, SK, and ZZ performed the experiments.

## Conflict of Interest Statement

The authors declare that the research was conducted in the absence of any commercial or financial relationships that could be construed as a potential conflict of interest.
